# Preoperative evaluation of femoral and tibial sagittal alignment in robotic-assisted and conventional total knee arthroplasty and consequences for practice

**DOI:** 10.1007/s00264-024-06229-x

**Published:** 2024-05-29

**Authors:** Yue Peng, Ran Ding, Ming Li, Guangxue Wang, Zikang Zhong, Lingbo Wei, Cheng Huang, Nianfei Zhang, Philippe Hernigou, Weiguo Wang

**Affiliations:** 1 China-Japan Friendship Hospital (Institute of Clinical Medical Sciences), Chinese Academy of Medical Sciences & Peking Union Medical College, Beijing, China; 2 Department of Orthopaedic Surgery, China-Japan Friendship Hospital, No.2 Yinghuayuan East Street, Chaoyang District, Beijing, 100029 China; 3 Peking University China-Japan Friendship School of Clinical Medicine, Beijing, China; 4 Peking Union Medical College Hospital, Chinese Academy of Medical Sciences and Peking Union Medical College (CAMS & PUMC), Beijing, China; 5 Department of Orthopaedic Surgery, University Paris East (UPEC), Hôpital Henri Mondor, Creteil, France

**Keywords:** Knee arthroplasty, Robotic surgery, Sagittal alignment, Angular differences

## Abstract

**Purpose:**

Robot-assisted total knee arthroplasty (TKA) was developed to improve the precision and accuracy of implant placement in conventional TKA. However, the angular differences between referenced axes in robot-assisted TKA and conventional TKA remain unclear. The aim of this study was to investigate the angular differences in sagittal alignment between robot-assisted TKA and conventional TKA for both the femur and the tibia and to discuss their clinical implications.

**Methods:**

We conducted a retrospective analysis of data from 100 patients (97 patients) who underwent computed tomography (CT) for Mako TKA. We measured the angle between the robot femoral axis (RFA) and conventional femoral axis (CFA) in the sagittal plane and the angle between the robot tibial axis (RTA) and the conventional tibial axis (CTA). Angles were compared between the sexes. Correlation analysis was conducted between the angles and height.

**Results:**

In the sagittal plane, the mean RFA-CFA angle was 2.2° ± 1.6°, and the mean RTA-CTA angle was 2.3° ± 1.6°. There were no significant differences between the two angles among males and females (p > 0.05). There was a correlation between the RFA-CFA angle and RTA-CTA angle (*p* < 0.001, r = 0.33), and there was a correlation between height and the combination of the RFA-CFA angle and RTA-CTA angle (*p* = 0.03, r = 0.22).

**Conclusion:**

There are angular differences between the axes referenced by robot-assisted TKA and those referenced by conventional TKA, which may be influenced by patient height. Correctly understanding these differences is crucial when evaluating the implant position and surgical outcomes after robot-assisted TKA. Furthermore, caution should be taken when assessing the flexion–extension angle of the knee since the angles displayed in the Mako system are different from the angles measured with intramedullary anatomical axes. After all, sagittal alignment principles differ between robot-assisted and conventional TKA; however, further studies are required to determine which principle is more appropriate or to modify these principles.

## Introduction

Total knee arthroplasty (TKA) is an effective surgical procedure for ameliorating severe osteoarthritis and other related conditions [1–3]. In recent years, robot-assisted TKA surgery has been recognized as an innovative approach for improving clinical outcomes [4, 5], and its clinical efficacy has been extensively evaluated [6, 7]. On the other hand, the difference between alignments of robot-assisted and conventional TKA procedures, which usually use an intramedullary jig system, has gradually become a concern [8–11].

The coronal alignment in TKA has gained considerable attention. There are many different alignment principles, such as mechanical alignment (MA), anatomic alignment (AA), restricted kinematic alignment (rKA), adjusted mechanical alignment (aMA), and functional alignment (FA) [12]. However, sagittal alignment in TKA has been somewhat overlooked. At the same time, it is crucial to perform TKA since the main movement of the knee joint is flexion and extension on the sagittal plane. Therefore, the restoration of sagittal anatomy is essential for optimal recovery of knee joint function.

Currently, robot-assisted TKA is becoming increasingly popular. With the application of CT scans and navigation, robot-assisted TKA is more advanced than X-ray-based conventional TKA, as is the case for dimensional reduction. In conventional TKAs, despite the inaccuracies and difficulties generated by the patient's position during frontal and lateral radiographs, two-dimensional X-rays are widely used before knee replacements to assess limb alignment, after which the implant position is targeted according to the alignment. Conversely, in robot-assisted TKA, with CT scans, the alignment of the limb is measured using a mathematical reconstructed model to calculate the coordinates of the axes of the femur and the tibia. This technique is more precise since it does not depend on the patient's position.

However, it is crucial to correctly understand the differences between alignment in robot-assisted and conventional intramedullary jig-TKAs, and this understanding should further improve the overall effect and long-term clinical outcome of TKA through relevant correction. These differences are more distinct in the sagittal plane than in the coronal plane and require special attention. Different reference axes may result in different amounts of bone resection; for precision-oriented surgical interventions using robotic systems, even minute variations in angles may result in alterations in the quantity of bone resected [5, 13]. Divergent femoral and tibial implantation schemes may also potentially influence the extent of bone resection [14]. Moreover, different prosthetic placement strategies, such as variations in femoral component height and the posterior slope of the tibial tray, can also affect surgical outcomes [15, 16]. Therefore, it is critical to understand the differences between the reference axes of the Mako robotic navigation system and those of conventional TKA surgery [11, 17, 18].

Conventional TKA utilizes the intramedullary axis as a reference for the femoral component position, while the Mako system used in robot-assisted TKA references the mechanical axis of the femur, which is defined as a vector from the hip centre to the knee centre. Changes in sagittal alignment have the potential to significantly alter knee kinematics, soft tissue tension, and implant longevity [19]. To our knowledge, previous studies have not fully investigated the angular differences between the referenced axes among robot-assisted TKA and conventional TKA. In this study, we therefore aimed to use the Mako system alongside other radiological measurement tools to quantify the angular difference between the reference axes of robot-assisted TKA and conventional TKA and to investigate the relationship between the angles and height so that the potential clinical implications can be discussed.

## Methods

We conducted a retrospective study in which data were collected from 97 patients (20 men and 77 women, Table 1) who received CT scans before TKA surgery at the China-Japan Friendship Hospital. The inclusion criteria for this study were patients who had severe osteoarthritis and required robot-assisted placement of a total knee prosthesis. Patients who met the following criteria were excluded: had a body mass greater than 40, were suspected of having infection, had neurological diseases, or were incapable of signing the consent form.

**Table 1:** Patient demographics

	Mean ± SD	Range
Age (years)	68.9 ± 7.6	33–83
Weight (kg)	68.4 ± 11.8	40.0–111.0
Height (cm)	161.3 ± 6.5	150.0–176.0
BMI* (kg/m2)	26.3 ± 3.8	16.0–38.0

^*^BMI body mass index

The CT scans were obtained by following the same protocols that correspond to the requirements of the Mako robotic TKA system (Mako, Stryker, Mahwah, NJ). For the CT scan, each patient was positioned head-first supine with a foam bolster placed below the contralateral unaffected knee to allow visualization of the affected knee during sagittal planning for 3D image acquisition. The foam bolster was then removed, scanning commenced with the lower limbs in a neutral position and the knees extended. Three sets of cuts passed through the hips, knees, and distal tibia-ankles. Stryker manufacturers did the 3D reconstruction of CT scans for surgical navigation. Two orthopaedic surgeons (PY and DR) measured the angles using Mimics, Version 18.0 (Materialise), and the mean values were calculated.

### For the femoral side

In the Mako system, the hip centre is defined as the centre of the femoral head, and the femur knee centre is defined as the most distal point of the trochlear groove. The robot femoral axis (RFA) used in the Mako system is identified as the line connecting the hip centre and the femur knee centre. The conventional femoral axis (CFA) is defined as the line that connects two distal femoral intramedullary centres (approximately 5–15 cm proximal to the joint line). We referenced the plane that was normal to the transepicondylar axis (TEA) as the sagittal plane. The RFA and CFA were projected on the sagittal plane, and the angle between the two axes was recorded as the sagittal RFA-CFA angle. If the mechanical axis was more extended than the anatomical axis, the sagittal RFA-CFA angle was recorded as positive.

### For the tibial side

**Fig. 1:**
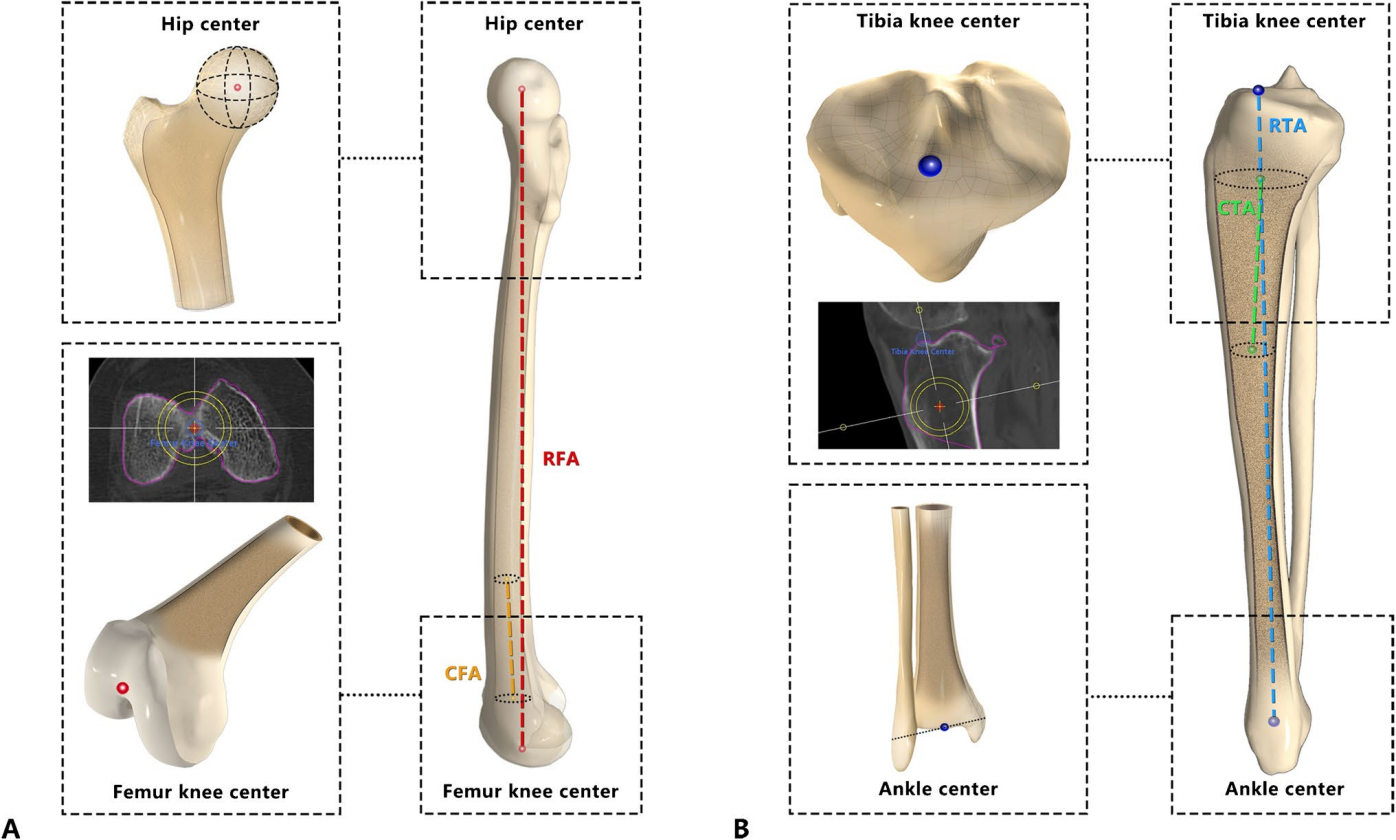
Axes of the lower limbs. Figure 1-**A** The hip centre is identified as the centre of the femoral head. The femur centre is identified as the most distal point of the trochlear groove. The CFA connects two distal intramedullary centres, and the RFA connects the hip centre and femur knee centre. Figure 1-**B** The tibial knee centre is identified as the exit point of the tibial shaft axis. The ankle centre is identified as the point located at 44% of the intermalleolar distance from the medial malleolus. The CTA connects two proximal intramedullary centres, and the RTA connects the tibial knee centre and ankle centre. RFA: robot femoral axis; CFA: conventional femoral axis; CTA: conventional tibial axis; RTA: robot tibia axis

**Fig. 2:**
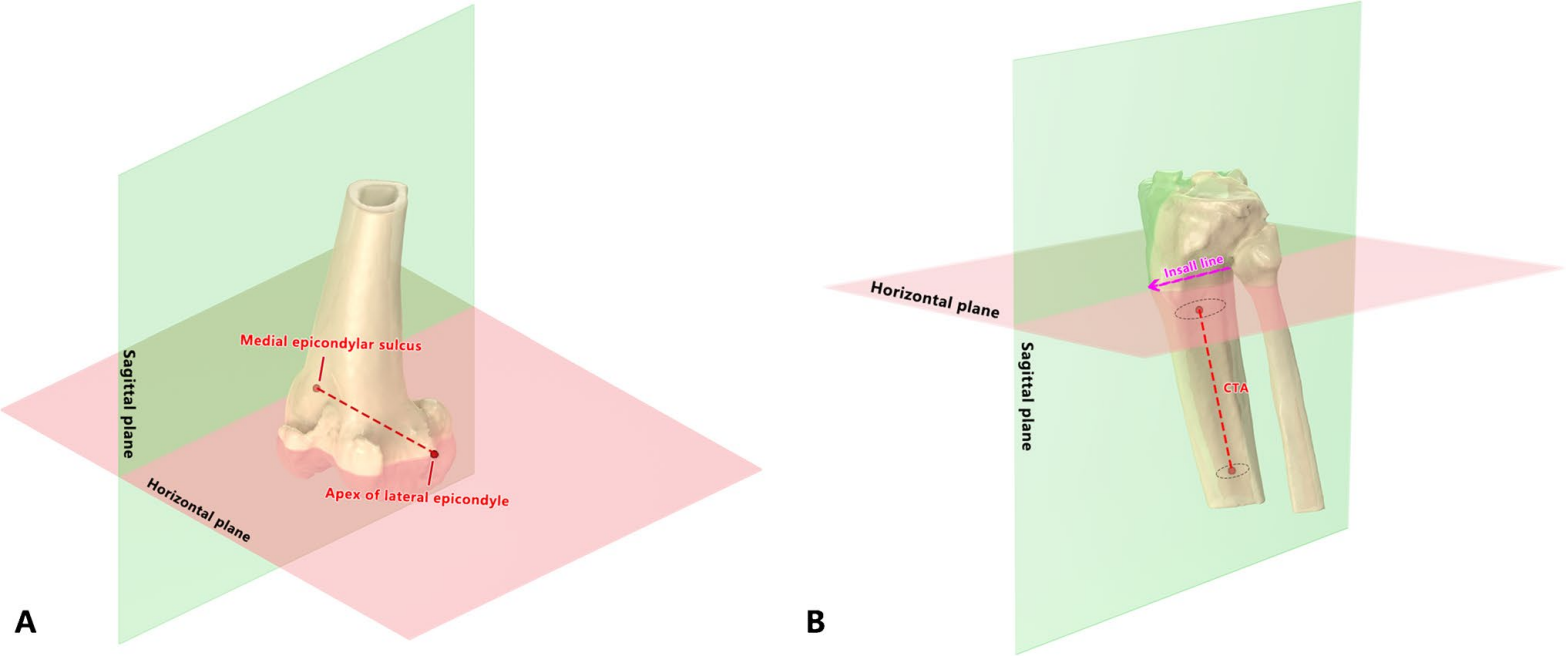
Sagittal plane definition. Figure 2-**A** The reference plane of the femoral side; the sagittal plane is perpendicular to the transepicondylar axis. Figure 2-**B** The reference planes of the tibial side, the horizontal plane perpendicular to the CTA, and the sagittal plane where the Insall line is located

In the Mako system, the tibial knee centre is the exit point of the tibial shaft axis in the coronal and sagittal planes. Here, we defined the robot tibia axis (RTA) as the line between the intermalleolar point located at 44% of the intermalleolar distance from the medial malleolus (which is also known as the ankle centre) and the tibial knee centre, and the conventional tibial axis (CTA) was defined as the line between two proximal intramedullary centres (approximately 5–15 cm distal to the joint line, Fig. 1). We referenced the plane that was normal to the CTA image as the transverse plane and projected the Insall line on the transverse plane; then, the plane that was perpendicular to the transverse plane and parallel to the projection of the Insall line was defined as the sagittal plane (Fig. 2). In this way, we measured the angular difference between the CTA and the RTA in the sagittal plane as the sagittal RTA-CTA angle. If the CTA was more extended than the RTA, the sagittal RTA-CTA angle was recorded as positive.

### Statistics

The demographic data and measurement results were analysed by using SPSS, version 24.0 (IBM). The intraclass correlation coefficient (ICC) was used to evaluate the agreement between the two observers. The means of angular differences between males and females were compared with the independent *t* test, and Pearson correlation analysis was performed between the sagittal RFA-CFA angle and the sagittal RTA-CTA angle and between their combination and height. The violin plot and scatter plot were drawn with GraphPad Prism, version 10.0.2 for Windows (GraphPad Software).

## Results

A total of 100 patients, including 97 patients (male to female ratio = 76:21) with 48 left knees and 52 right knees, were included in the study.

**Fig. 3:**
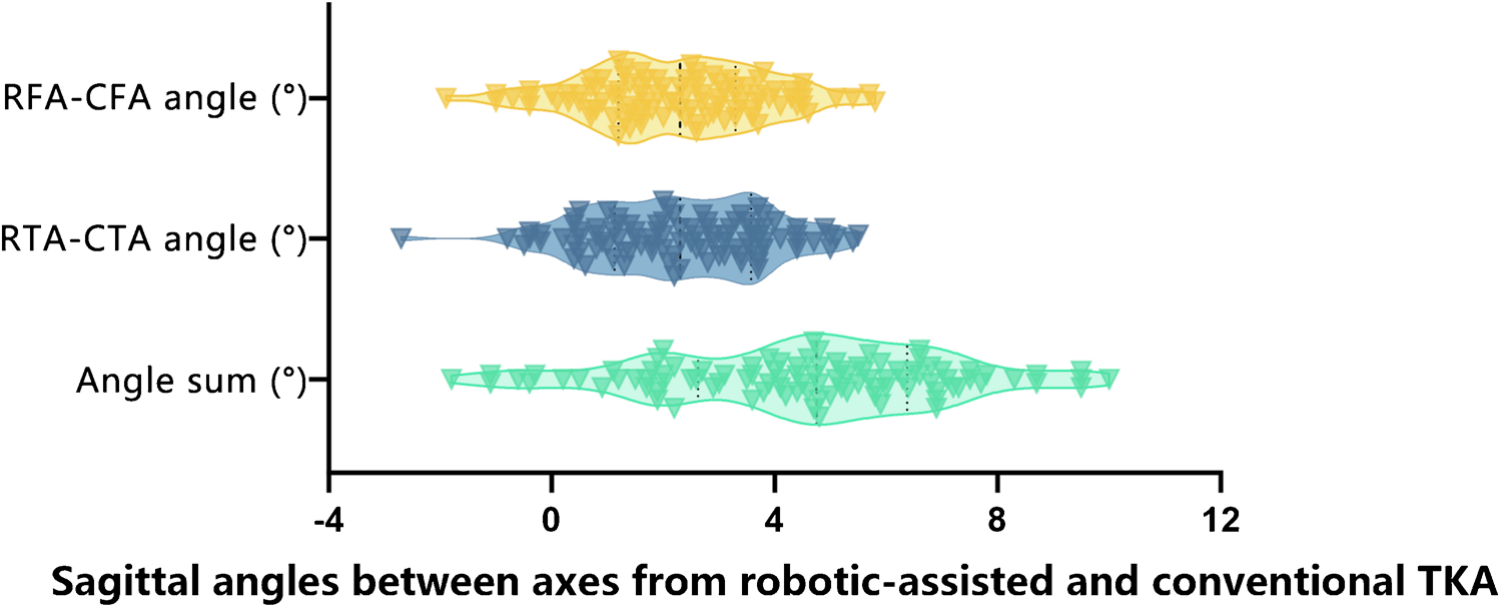
Violin plot of different angles

In terms of the femoral side at the sagittal plane, in 91/100 knees, the RFA was at the most extended position to the CFA, and the mean RFA-CFA angle was 2.2° ± 1.6° (average ± standard deviation), ranging from -1.9° to 5.8°; 34% of the included knees had RFA-CFA angles greater than 3°. Regarding the tibial side at the sagittal plane, in 93/100 knees, the CTA was more extended than was the RTA at the sagittal plane, and the mean RTA-CTA angle was 2.3° ± 1.6°, ranging from -2.7° to 5.5° (Table 2); 37% of the knees had an RTA-CTA angle greater than 3°. The mean value of the combination of the RFA-CFA angle and the RTA-CTA angle (angle sum) was 4.5° ± 2.6°, ranging from -1.8° to 10.0°. A violin plot of different angles is shown in Fig. 3. The ICCs of the RFA-CFA angle and RTA-CTA angle between the two surgeons were 0.92 and 0.94, respectively.

**Table 2:** Measurements of different angles

	Mean ± SD	Range
Sagittal RFA-CFA angle	2.2° ± 1.6°	-1.9° to 5.8°
Sagittal RTA-CTA angle	2.3° ± 1.6°	-2.7° to 5.5°
Angle sum	4.5° ± 2.6°	-1.8° to 10.0°

**Fig. 4:**
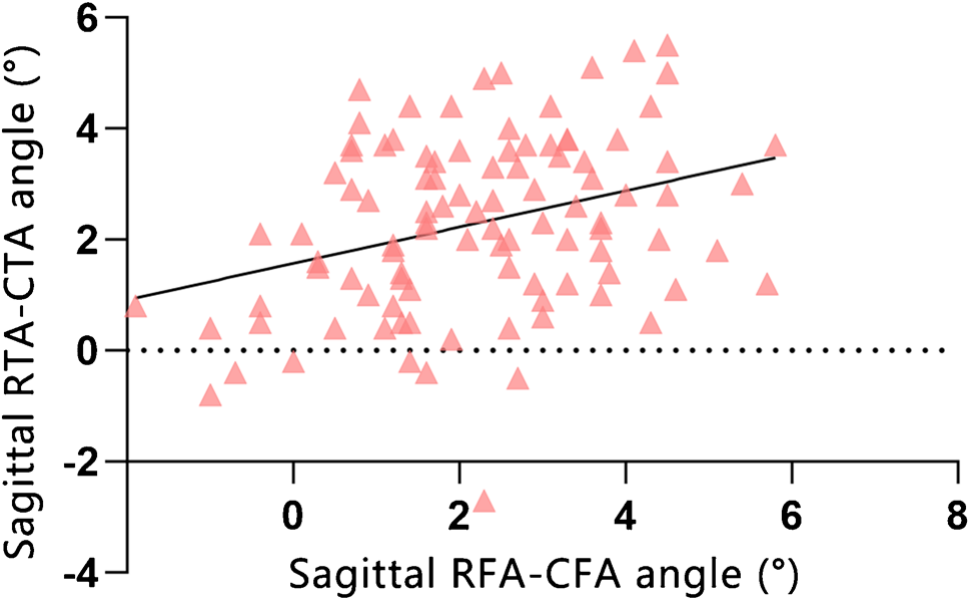
The correlation between the RFA-CFA angle and RTA-CTA angle

**Fig. 5:**
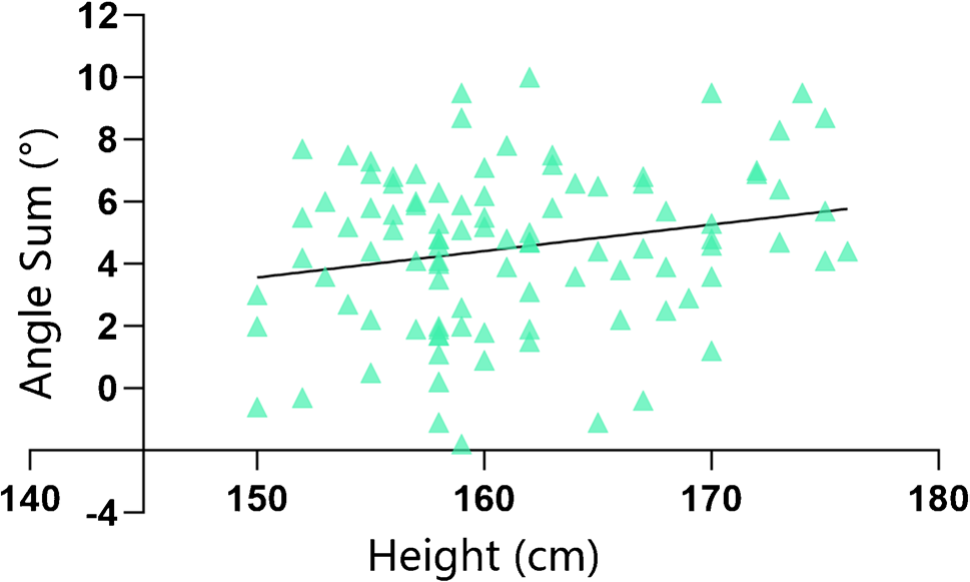
The correlation between height and the combination of the RFA-CFA angle and RTA-CTA angle

In the sagittal plane, there were no significant differences between the two angles between males and females (RFA-CFA angle *p* = 0.66, RTA-CTA angle *p* = 0.69). There was a correlation between the sagittal RFA-CFA angle and the sagittal RTA-CTA angle (*p* < 0.001, r = 0.33; Fig. 4), and there was also a correlation between height and the combination of the RFA-CFA angle and the RTA-CTA angle; however, the correlation was weak (*p* = 0.03, r = 0.22; Fig. 5).

## Discussion

In this study, we found angular disparity in the sagittal plane between the lower limb anatomical axis, which was referenced by conventional TKA, and the mechanical axis, which was referenced by robot-assisted TKA. On the femoral side, most of the RFAs were located more extended to the CFAs. On the tibial side, most of the CTAs in the sagittal plane were also more extended than were the RTAs. These angular values warrant attention because they may impact the placement of knee joint prostheses during TKA surgery, intraoperative navigation system flexion–extension angle display, and postoperative evaluation.

Regarding our femoral measurements, the data from this study corroborate previous research since most of the robot femoral axes were more extended to the conventional femoral axes [20]. The mild differences between our study and previous studies are possibly due to two factors. First, prior research has used the midpoint of the medullary canal at the femoral diaphysis, whereas this study used a location approximately 5–15 cm proximal to the joint line. Hence, the disparities in results between these measurement approaches may be attributed to differences in anatomical location. Second, prior research has indicated that individuals of Asian descent exhibit a larger posterior bow of the femur. Chen et al. described Asian women with increased anterior bowing in particular [21], and Hood et al. reported that patients of Asian descent are more likely to have a distal femoral flexion (DFF) angle greater than 5°[22]. All of these factors may contribute to the observed angular deviation between the CFA and RFA.

From the surgeons’ perspective, the RFA-CFA angle can influence the position of femoral implants. The optimal femoral component on the sagittal plane is considered the one that has the largest size without causing notching on the anterior face of the femur. According to our study, most patients have a more extended RFA compared to the CFA; to avoid notching, surgeons may consider flexing the femoral component during implant planning if the difference between the CFA and the RFA is significant. By doing so, the component will move to a better position for a more uniform bone-component interface.

From a prosthesis design perspective, the sagittal plane angular difference between the CFA and RFA minimally affects a single-radius femoral component, whereas multiple-radius femoral components may lead to an extension of the femoral component if placed according to the robot axis, thereby impacting the balance of the flexion–extension gap and potentially increasing the risk of the notch phenomenon and patellar tracking problems. Tadashi et al. discovered that each intentional 2° increase in flexion of the femoral component led to a decrease of 1 mm in flexion space, which shows that adjusting the flexion gap by sagittal flexion of the femoral component can be a useful complementary option for avoiding excessive elevation of the joint line [23].

Within tibial measurements, we ascertained in this study that the tibial axis exhibits an angular deviation in the sagittal plane when measured by both robot-assisted and conventional methods. Since direct determination of the ankle centre in the sagittal plane is not feasible using the Mako navigation system, RTA was employed to approximate the tibial anatomical axis as defined by the Mako system. Our results revealed that the RTA typically aligned posterior to the CTA. From the perspective of intraoperative navigation, the presence of angular deviation implies that the posterior slope planned during Mako surgery may be smaller than that planned for the conventional method, which is consistent with previous research findings [9]. This could decrease overall bone resection or cause a relatively tight flexion gap. However, it must also be considered that the radiological measurement of the posterior slope is not the true physiological posterior slope, which includes the thickness of the meniscal posterior horn and is, therefore, lower than the radiological measurement. All these data influence the overall bone resection and/or the final tight flexion gap.

Correctly understanding the differences in reference axes between robot-assisted and conventional TKAs is crucial when comparing surgical outcomes and evaluating postoperative implant positions. According to the results of our study, objective differences exist in the reference axes between robot-assisted TKA and conventional techniques. The robot reference axis is established from a comprehensive global perspective. In contrast, conventional TKA only references the intramedullary axes in the distal femur and the extramedullary axes on the proximal tibia, which can be uncertain. Furthermore, lateral knee radiography is commonly used during postoperative assessment, in which the reference axes can only be located in the distal femur and proximal tibia.

Furthermore, careful consideration is required regarding the flexion–extension angles used in Mako TKA. Our research suggested that compared to traditional TKA techniques, robot-assisted TKA may result in a more extended femoral component and a more anteriorly tilted tibial component; when these conditions exist simultaneously, a significant difference can occur. Our results also imply that when the Mako system indicates that the knee is fully extended (0°), it may represent overextension relative to the anatomical axes of the femur and tibia. Similarly, when surgeons subjectively recognize that the knee is fully extended, the Mako system can still indicate some degree of flexion deformity. Therefore, caution must be taken to avoid the occurrence of knee joint overextension in robot-assisted TKA, especially in patients with prominent femoral and/or tibial bowing.

Moreover, considerations regarding the sagittal alignment of the mechanical alignment (MA) and anatomical alignment (AA) are crucial. While coronal alignment principles have received great attention and have been the topic of discussion in modern TKA, sagittal alignment has been relatively understudied. Robot-assisted TKA based on CT reconstructions can be considered to involve mechanical alignment (with the femoral side representing a standard MA and the tibial side approximating the MA), whereas conventional TKA involves alignment of the AA. There is no doubt that discrepancies between these alignment principles exist in the sagittal plane; however, further investigations are required to investigate whether these differences affect knee joint kinetics and to determine which alignment principle is more appropriate or whether there are any modifications among these principles.

In addition, our study indicated that height can be a simpler predictor of the sum of the sagittal RFA-CFA angle and the RTA-CTA angle. We found that the sums of the angles were greater when patients were taller. Previous studies have shown that the femur sagittal plane RFA‒CFA angle is positively correlated with femoral neck anteversion and femoral bow [24]. However, due to the limited scan range of the Mako-CT scanner and the large scan slice thickness of the hip joint, it may not be possible to obtain accurate data on femoral neck anteversion and femoral bow from the Mako-CT scanner. Therefore, height can be used as a simpler indicator to predict the differences between the referenced axes of robot-assisted TKA and conventional TKA.

Despite these findings, this study is not devoid of limitations. First, Mako-CT scanning cannot encompass the entire length of the femur and tibia and therefore may overlook certain anatomical anomalies. Second, this was a single-centre study, which limits the robustness of the evidence to broader populations. Third, the potential influence of the measured angles on surgical outcomes requires further substantiation through rigorous controlled studies. Moreover, intraobserver and interobserver analyses were carried out by two people in a cohort of 97 patients. Notably, the alignment measurements were also performed on non-weight-bearing CT scans. This does not change bone landmarks but does not allow us to know the exact position of the knee when the patient is walking, mainly if the hip is stiff in a fixed abnormal rotation position. In the future, artificial intelligence could improve the precision of acquisitions by more precisely identifying anatomical coordinates and analysing the patient's forward gait to match the gait with the measurements made by the CT scan. Nevertheless, the measurements carried out by this study on the sagittal plane are the first data that can serve as a reference for future work and for a better understanding of all the publications on robotic surgery [25–29].

## Conclusion

In the sagittal alignment of TKAs, angular differences objectively exist between the axes referenced by robot-assisted TKA and those referenced by conventional TKA, which may be predicted by the height of patients. Correctly understanding these differences is crucial when comparing surgical outcomes between conventional and robot-assisted TKA and evaluating the implant position after robot-assisted TKA since these angular differences can lead to excessive extension of the femoral components and decrease the posterior slope of the tibial platform. On the other hand, caution should be taken when evaluating the flexion–extension angle of the knee since the angles displayed in the Mako system are different from the angles measured with intramedullary anatomical axes. The principles of sagittal alignment differ between the MA and AA, which are used in robot-assisted TKA and conventional TKA, respectively. Further studies are required to determine which principle is more appropriate or to modify these principles.

## Data Availability

The data that support the findings of this study are available on request from the corresponding author.
